# Cropping With Slag to Address Soil, Environment, and Food Security

**DOI:** 10.3389/fmicb.2019.01320

**Published:** 2019-06-18

**Authors:** Suvendu Das, Gil Won Kim, Hyun Young Hwang, Pankaj Prakash Verma, Pil Joo Kim

**Affiliations:** ^1^Institute of Agriculture and Life Sciences, Gyeongsang National University, Jinju, South Korea; ^2^Hawkesbury Institute for the Environment, Western Sydney University, Penrith, NSW, Australia; ^3^Division of Applied Life Science, Gyeongsang National University, Jinju, South Korea

**Keywords:** microbial dynamics, silicate fertilization, slag, greenhouse gas emissions, carbon sequestration

## Abstract

The effective utilization of slag fertilizer in agriculture to neutralize soil acidity, improve crop productivity, mitigate greenhouse gas emissions, and stabilize heavy metals in contaminated soils turns it into a high value added product in sustainable agriculture. These effects could be due to the shift in microbial metabolism and/or modification of microbial habitats. At the system level, soil microorganisms play an integral role in virtually all ecosystem processes. There is a growing interest to reveal the underlying mechanisms of slag-microbe interactions and the contribution of soil biota to ecosystem functioning. In this perspective, we discuss the possible driving mechanisms of slag-microbe interactions in soil and how these slag-microbe interactions can affect crop yield, greenhouse gas emissions, soil carbon sequestration, and heavy metal stabilization in contaminated soils. In addition, we discuss the problems and environmental concerns in using slag in agriculture. Emphasis has been given for further research to validate the proposed mechanisms associated with slag-microbe interactions for increasing soil quality, crop productivity, and mitigating environmental consequences. While evaluating the slag amendment, effects on agriculture and environment, the potential risks, socio-economics, techno-economics, and ethics should be assessed.

## Introduction

Over the past decades, with the rapid growth of industrialization, the higher volume of byproducts (slag) generated from iron/steel production draw attention to the need for its recycling in an increasingly efficient way. With the increase in population, the available land to dispose of large amounts of slag in landfill sites is reduced and the disposal cost is becoming increasingly higher. Moreover, the land filled with disposed slag has become an important source of pollution of air, water, and soil, which further adversely affects vegetation and human health (Branca and Colla, 2012). The entry of heavy metals/metalloids into the food chain is a critical issue of current public health (Chand et al., 2015). From the perspective of natural resource conservation, environmental protection, and human health safety measures, recycling of slag has drawn the attention of scientists, environmentalists, and policymakers in recent years. The increase of slag recovery and use in different fields of application, such as agriculture, is an imperative way for sustainable development (Ito, 2015).

Slag consists mostly of mixed oxides of elements such as silicon, sulfur, phosphorus, and aluminum, and products formed in their reactions with furnace linings and fluxing substances such as limestone (Yildirim and Prezzi, 2011; Piatak et al., 2015). Since slag is rich in lime (CaO), silicic acid (SiO_2_), phosphoric acid (P_2_O_5_), magnesia (MgO), Mn, and Fe, these properties of the slag can be exploited to make use of fertilizer (Ito, 2015). Notably, steel-making slag and blast furnace slag have been extensively utilized as raw materials for fertilizer production, mostly in Japan, Korea, and China. Fertilizers made of slag are categorized as slag silicate fertilizer, lime fertilizer, slag phosphate fertilizer, and iron matter of special fertilizer (Ito, 2015). In recent years, several studies have revealed that the slag-based fertilizer amendment in agriculture has great promise to improve crop productivity (White et al., 2017; Gwon et al., 2018), alleviate soil acidification (Ning et al., 2016), mitigate greenhouse gas (GHG) emissions (Wang et al., 2015; Gwon et al., 2018), and stabilize heavy metals in contaminated soils (Ning et al., 2016), which turns it into a high value added product for sustainable agriculture. These beneficial effects of slag fertilization largely rely on the changes in soil microbial habitats and microbial activities. In fact, at the system level, soil microorganisms play a vital role in virtually all ecosystem processes and provide ecosystem services crucial for the maintenance of soil quality and productivity (Das et al., 2017). In this perspective, we discuss the driving mechanisms of slag-microbe interactions in soil, and slag-microbe interaction effects on crop yield, greenhouse gas reduction, soil carbon storage, and heavy metal stabilization in contaminated soils. Lastly, we discuss environmental concerns about the use of slag in agriculture and the future perspectives.

## Driving Mechanisms of Slag-Microbe Interactions in Soil

**Figure 1:**
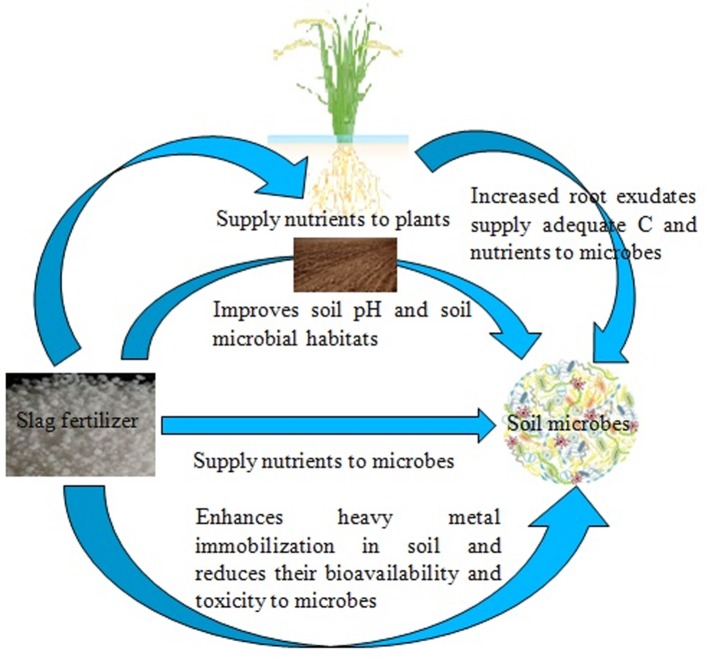
The proposed mechanism of slag-microbe interactions in soil.

**Figure 2:**
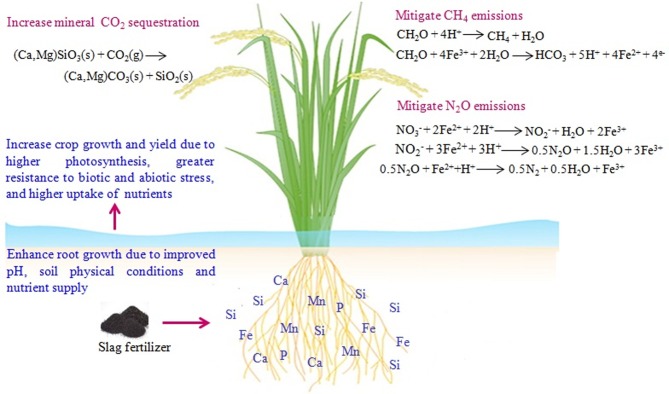
Summary of potential effects of slag on crop plants.

The shift in soil microbial community and activities in response to slag fertilizer amendment may depend on the type of slag fertilizer (e.g., silicate fertilizer, lime fertilizer, slag phosphate fertilizer, and iron matter of special fertilizer), which modify soil properties and soil microbial habitats. With advances in omic techniques, soil microbial communities and community-level molecular characteristics have been exploited as early indicators of ecosystem processes for sustainable soil management and agricultural productivity (Shokralla et al., 2012). In recent years, extensive research has been conducted to obtain a mechanistic understanding of the contribution of microbial communities to ecosystem functioning under various agronomic management practices. Unfortunately, few studies have focused specifically on understanding the changes in soil microbial community and function under slag fertilizer amendment in cropping systems. Since the mechanisms of slag-microbe interactions in soil are still not clear, this perspective focuses on the synthesis of several possible mechanisms based on published research. The influence of slag fertilizer on the soil microbiome are diverse and the possible mechanisms of slag-microbes interactions can be as follows: (1) slag fertilizer supplies nutrients not only to the plant but also to soil microorganisms; (2) slag fertilizer modifies soil microbial habitats by improving soil properties (e.g., increasing soil pH) (Gwon et al., 2018), which is essential for nutrient mobilization and microbial growth; (3) silicate fertilizer increases plant photosynthesis (Detmann et al., 2012) and likely increases belowground carbon allocation through root exudates, which eventually triggers soil microbial proliferation and activities; and (4) steel slag enhances heavy metal immobilization in soil (Ning et al., 2016) and thus reduces their bioavailability and toxicity to microbes. Besides, the slag fertilizer amendment may induce changes in soil enzyme activities that affect soil nutrient mobilization and microbial dynamics. In Figure 1, we show the proposed mechanism of slag-microbe interactions in soil. The proposed mechanisms of slag-microbe interactions need to be experimentally verified and intensive research needs to be conducted to explore the microbial role in soil processes and agricultural productivity. The potential effects of slag on crop plants have been described in the separate subheading and also shown in Figure 2.

## Slag-Microbe Interaction Effects on Crop Yield

Among fertilizers made from slag, the use of silicate fertilizer, particularly in rice cropping systems has been gaining awareness and demand (Meharg and Meharg, 2015). Rice is a high Si accumulating plant. Intensive rice cultivation to meet the growing food demand chronically depletes Si from soil, thus degrades soil quality and decreases the crop yield (Branca and Colla, 2012). This necessitates silicate fertilizer amendment in rice cropping systems for sustainable rice cultivation. Studies have indicated an increase (0.16–47.2%) in rice grain yield in lowland rice fields following the addition of slag-based silica fertilizer (Supplementary Table S1). The increase in grain yield, however, mostly depends on slag type, application rate, soil type, and agronomic management. Ali et al. (2009a) reported that the silicate fertilizer amendment in no-tillage submerged paddies could improve the crop yield up to 47.2%. Wang et al. (2015) suggested that the silicate fertilization is not significantly effective in improving the rice yield. Higher grain yields in response to silicate fertilization could be attributed to the fact that Si preferentially deposits in the epidermal cell wall and increases physical strength of leaves and leaf-sheaths and help plants to sustain yield by counteracting various biotic and abiotic stresses and increasing plant biomass (Luyckx et al., 2017). Besides silicate fertilizer, lime fertilizer has been widely used in acidic soil to neutralize the soil, which helps plants to protect themselves against soil pathogens. In addition, Ca content in slag fertilizer makes the roots strong and promotes the absorption of K, which is important for plant growth. Slag phosphate fertilizer has been used to provide adequate phosphorus to the plant, which improves plant growth and fruiting. Likewise, the iron matter of special fertilizer has been used to mitigate the toxicity of heavy metals in soil as well as in the plant. Noteworthy, the increased yield under slag fertilization is largely regulated by microbial decomposition of organic matter and nutrient mobilization. It can be postulated that slag fertilizer amendment not only increases soil nutrients *per se,* but also enriches soil microorganisms that have a beneficial role in nutrient mobilization (e.g., carbon and nitrogen mineralization, phosphorus solubilization, nitrogen fixation, etc.). Identification and elucidation of functional roles of keystone soil microbes that sustain plant health and productivity under slag fertilization could provide a technological breakthrough for a sustainable use of slag in agricultural productivity.

## Slag-Microbe Interaction Effects on Greenhouse Gas Emissions

Agriculture significantly contributes to the emission of methane (CH_4_) and nitrous oxide (N_2_O), which are two of the most important greenhouse gases responsible for global warming (Das and Adhya, 2014). Methane emission from soils is regulated by CH_4−_producing archaea, i.e., methanogens, and CH_4_-consuming bacteria, i.e., methanotrophs, while N_2_O emission is mostly regulated by nitrifying and denitrifying bacteria (Singh et al., 2010). Soil amendment that reduces methanogen abundance and activity, and/or increases methanotroph abundance and activity could be effective to mitigate CH_4_ emissions from the soil. Slag fertilizers, in particular, iron/steel slag fertilizers are rich in iron. Iron acts as an alternative electron acceptor in anoxic soil and its application decreases CH_4_ emissions by stimulating iron-reducing bacteria at the expense of methanogens (Gwon et al., 2018). Ali et al. (2009b) showed that 4 mol of Fe^3+^ prevent the generation of 1 mol of CH_4_. Moreover, silicate fertilizer amendment can increase root biomass and O_2_ transport from the plant to root by enlarging arenchyma gas channels (Liang et al., 2007), which in turn suppresses CH_4_ production and stimulates CH_4_ oxidation. Studies conducted in Korea, Japan, China, Indonesia, and Bangladesh indicated the potential of slag fertilizer amendment to decrease CH_4_ emissions by 0.6–56.0% from lowland rice paddies (Supplementary Table S1). The extent of CH_4_ emissions reduction depended on the slag fertilizer type, rate of application, soil type, and agronomic practices (Supplementary Table S1). Wang et al. (2018a) showed that the application of slag fertilizer (8 Mg ha^−1^) with biochar (8 Mg ha^−1^) reduced CH_4_ emission up to 38.6% in early rice in China; however, Lee et al. (2012) reported that the silicate fertilization is not effective in reducing CH_4_ emissions in green manure amended paddy soils probably due to the enhanced decomposition of added organic matter by the silicate liming effect. Elucidation of methanogen and methanotroph diversity and their functional changes in response to slag fertilizer amendment will improve our mechanistic understanding of CH_4_ dynamics in relation to slag fertilization.

Unlike CH_4_ emissions, the slag fertilizer effects on N_2_O emissions from rice cropping systems are contradictory. Some studies suggest slag fertilizer decreases N_2_O emissions (Susilawati et al., 2015; Wang et al., 2015), while other studies suggest slag fertilizer increases N_2_O emissions (Huang et al., 2009; Liu et al., 2012). Wang et al. (2015) indicated that a 99% reduction in N_2_O emissions could be achieved in an intermittent irrigated rice paddy using silicate fertilizer at the rate of 8 Mg ha^−1^. The decrease in N_2_O emissions have been attributed to lower N availability and higher Fe availability with Si fertilization. Iron oxidation coupled to denitrification can occur in anoxic soils, which can lead to N_2_O production (Melton et al., 2014); however, under conditions where Fe is highly available, the electrons donated by Fe(II) exceed the electron demand for N_2_O production, which leads to complete denitrification to N_2_ and thus a suppression of N_2_O emissions (Wang et al., 2016). Increases in N_2_O emissions with Si fertilization have been attributed to: (1) Si fertilization acting to lower soil C decomposition, which would alleviate immobilization of fertilizer N thereby making more mineral N available to nitrification and denitrification; and (2) Si fertilization improving soil pH and Eh, which are two factors important to N_2_O emissions (Huang et al., 2009; Liu et al., 2012). In a recent study, Song et al. (2017) reported that the silicate fertilizer amendment significantly decreased denitrification potential and *nirS* and *nirK* gene abundance in paddy soils. Owing to the contradictory results, the mechanism underlying N_2_O emissions and changes in the genetic potential of nitrifying and denitrifying bacteria under slag fertilization needs further investigation.

## Slag-Microbe Interaction Effects on Soil Carbon Storage

Carbon dioxide sequestration in soils is well recognized as an avenue to mitigate climate change. Mineral carbonation of CO_2_ (mineral CO_2_ sequestration) occurs spontaneously on geological time scales and has a high potential for CO_2_ sequestration (Oelkers et al., 2008). It typically involves the dissolution of silicate minerals and subsequent precipitation of stable carbonate minerals (e.g., CaCO_3_, MgCO_3_, and FeCO_3_). Mineral carbonation reactions require combining CO_2_ with metals to form stable carbonate minerals. With few exceptions, the required metals are divalent cations, including Ca^2+^, Mg^2+^, and Fe^2+^, and the most abundant cation source are silicate minerals. Although mineral carbonation is thermodynamically favorable, it proceeds very slowly (Oelkers et al., 2008). Research is going on worldwide to enhance mineral weathering processes and to accelerate mineral carbonation reactions (Salek et al., 2013). There are only few reports concerning the effects of the slag fertilizer amendment on carbon sequestration in cropping systems. Wang et al. (2018) reported that the addition of steel slag and biochar in subtropical paddy fields could decrease active SOC pools and enhance soil C sequestration only in the early crop, but not the late crop. Since slag fertilizers are a rich source of silicon minerals and alkaline in nature, their application in agricultural soil may potentially increase soil carbon sequestration. The use of slag fertilizer instead of agricultural lime (limestone) to increase soil pH would eliminate the dissolution of lime as an important source of agricultural CO_2_ emissions. It is well recognized that the enzyme carbonic anhydrase (CA) participates in silicate weathering and carbonate formation and thus plays an important role in the biomemetic CO_2_ sequestration (Bose and Satyanarayana, 2017). Bio-inoculation of bacteria possessing CA activity in slag fertilized agricultural systems could accelerate silicate weathering and enhance CO_2_ sequestration. Likewise, the introduction of plant growth promoting bacteria possessing CA activity in agriculture could have the dual benefit of increased crop yield and CO_2_ sequestration. In a recent review it is postulated that farming with rock could have a great promise in sequestering carbon in soils (Beerling et al., 2018). There is an urgent need to evaluate the fate of soil carbon and carbon sequestration potential of slag fertilizer in field conditions.

## Slag-Microbe Interaction Effects on Heavy Metal Stabilization in Contaminated Soils

The stabilization technique aims at reducing heavy metal and metalloid (e.g., As, Cr, Cu, Pb, Cd, and Zn) bioavailability in contaminated soil. The technique is based on amendments to change the soil physicochemical properties through adsorption, precipitation, ion-exchange techniques, redox potential technology, and pH control technology that change the existing forms and speciation of heavy metals/metalloids and thus, reduce their toxicity (Mosa et al., 2016). There are several examples, as follows: as can be stabilized by sorption on Fe oxyhydroxide and also by the formation of amorphous Fe(III) arsenates; Cr can be stabilized by the reduction from more mobile and toxic Cr(VI) to less toxic and stable Cr (III); Cu can be stabilized by precipitation of Cu carbonates and oxyhydroxides, iron exchange and formation of ternary cation-anion complexes on the surface of Fe and Al oxyhydroxides; and Pb and Zn can be immobilized by phosphorus amendments (Branca and Colla, 2012). The slag fertilizer amendment markedly affects the soil solution composition through acid–base, precipitation, and sorption reactions. Owing to its suitable chemical and mineralogical properties, slag fertilizer has been used as a stabilizing agent to minimize metal and metalloid contamination in soil (Ning et al., 2016). Moreover, the adequate Si supply through slag silicate fertilizer amendment causes competitive inhibition of As(III) uptake by crop plants (Meharg and Meharg, 2015). The effects of slag fertilizer amendment on the biogeochemical cycling of soil elements that are regulated by soil microbes need to be investigated. A combination of slag fertilizer and microbial remediation strategies could be proposed for effective remediation of soil contaminants.

## Environmental Concerns About the Use of Slag in Agriculture

The main concerns regarding the use of slag in agriculture are the potential for heavy metal accumulation in soil and the risks related to liming of soil (Chand et al., 2015). Slags contain traces of heavy metals, but the concentrations of heavy metals might not be enough to pose environmental risks (Gwon et al., 2018); however, it is believed that the long-term application of slag fertilizer in agriculture may accumulate heavy metals/metalloids in soil and may cause health risks. Several studies reveal that metal contamination in soil and metal uptake by plants are not adversely affected by short-term slag fertilizer amendment in cropping systems (Ali et al., 2008, Gwon et al., 2018). In addition, long-term experiments in Germany showed that steel slag fertilizer amendment did not increase bio-available Cr content in soil and Cr uptake by plants (Hiltunen and Hiltunen, 2004). Kuhn et al. (2006), however, revealed that the long-term application of converter slag significantly increased Cr and V contents in the cultivated layer of soil. For a better understanding of the long-term effects of the slag fertilizer amendment in agriculture, further research under diverse soil types and agronomic management practices need to be carried out. Due to the high reactivity of CaO and MgO and high pH (i.e., 12.5) of Ca(OH)_2_, repeated application of slag may make the soil excessively alkaline, which may decrease the bioavailability and uptake of macronutrients such as P and micronutrients such as Fe, Cu, and Zn by the plant and likely hinder plant growth and productivity (Chand et al., 2015). Another demerit of slag fertilizer is that it contains small proportions of N and K, and P (in some slag fertilizer), which are essential nutrients for plant growth. Therefore, slag fertilizer should be applied together with a chemical fertilizer that contains adequate amounts of N, P, and K.

## Conclusions and Future Directions

With the rapid increase in steel production, steel industries are under pressure for effective and eco-friendly recycling of slag. While in the past, steel-making processes were exclusively designed for the production of specific quality and quantities of iron and steel, one of today’s goals for steel making is to design and develop technologies to produce high-quality slag according to the market requirements. Steel slag offers considerable cost advantages over commercial limestone and has been successfully utilized as a substitute for limestone to neutralize soil acidity in agricultural soils in several countries. Owing to its high Si content, the use of slag as silicate fertilizer is gaining demand. The term “slag” is used in the specifications of slag silicate fertilizer and slag phosphate fertilizer in the Fertilizer Control Law. The slag can be mixed with livestock wastes to make compost, so that both slag and livestock waste can be effectively utilized in agriculture. However, to secure the reliability of the slag as fertilizer, it is quite necessary to conform to the regulations on hazardous heavy metals provided by the Fertilizer Control Law and the soil environmental standards provided by the Basic Law for Environmental Pollution Control.

Understanding the effects of slag fertilizer on soil microbial communities and functions is essential to address some critical agro-environmental issues, such as whether the slag fertilizer amendment would be useful to increase crop productivity, reduce GHG emissions, increase soil carbon sequestration, and stabilize heavy metals in contaminated soils. The recent advances in omic techniques, e.g., high-throughput sequencing, metatranscriptomic analysis, and DNA/RNA-based stable isotope probing (SIP) will no doubt be imperative to uncover the hidden dimensions of slag-microbe interactions in ecosystem functioning.

## Author Contributions

SD wrote the manuscript. All authors contributed to the intellectual input and provided assistance to the manuscript preparation.

### Conflict of Interest Statement

The authors declare that the research was conducted in the absence of any commercial or financial relationships that could be construed as a potential conflict of interest.
