# Supplementary material for: Cropping With Slag to Address Soil, Environment, and Food Security
Source: Front Microbiol. 2019 Jun 18;10:1320. doi: 10.3389/fmicb.2019.01320 (PMC6592145; doi:10.3389/fmicb.2019.01320)
Supplement: Supplementary file 1 [file Data_Sheet_1.docx]

**Supplementary Table 1.** Summary of the slag-based silicate fertilizer effects on CH_4_ emission, N_2_O emission, SOC, and crop yield in rice cropping systems

| Slag type | Rate of application | Study duration | Cropping system | Soil type | Country | CH_4_ emission | | N_2_O emission | | SOC | | Yield | | Reference |
| --- | --- | --- | --- | --- | --- | --- | --- | --- | --- | --- | --- | --- | --- | --- |
|  |  |  |  |  |  | Control | Slag | Control | Slag | Control | Slag | Control | Slag |  |
| Silicate iron slag | 4 Mg ha^-1^ | 2 years (2006-2007) | Rice (Continuous irrigation) | Typic Haplaquepts  (Clay loam) | South Korea | 36.5 –38.1 g m^-2^ | 30.6 –30.5 g m^-2^  (-16-20%) | - | - | - | - | 6.22 Mg ha^-1^ | 7.03 – 7.34 Mg ha^-1^ (13-18%) | Ali et al., 2008 |
| Silicate fertilizer (granular) | 4 Mg ha^-1^ | 1 year (2007) | Rice (Continuous irrigation) | Typic Haplaquepts  (Clay loam) | South Korea | no-tillage  27.9 g m^-2^ | 18.0 g m^-2^  (-35.5%) | - | - | - | - | 6.42 Mg ha^-1^ | 9.45 Mg ha^-1^ (47.2%) | Ali et al., 2009 |
| LD slag | 2 Mg ha^-1^ | 1 year (2016) | Rice (Continuous irrigation) | Typic Haplaquepts  (Clay loam) | South Korea | 583 kg ha^-1^ | 458 kg ha^-1^  (-21.4%) | - | - | - | - | 5.7 g pot^-1^ | 6.3 g pot^-1^  (10.5%) | Gwon et al., 2018 |
| Silicate fertilizer (granular) | 2.3  Mg ha^-1^ | 1 year (2008) | Rice (Continuous irrigation) | Typic Haplaquepts  (Clay loam) | South Korea | Without Gm  488 kg ha^-1^ | 417 kg ha^-1^  (-14.5%) | - | - | - | - | 3.9 Mg ha^-1^ | 4.5 Mg ha^-1^  (15.4%) | Lee et al., 2012 |
| Silicate fertilizer (granular) | 2.3  Mg ha^-1^ | 1 year (2008) | Rice (Continuous irrigation) | Typic Haplaquepts  (Clay loam) | South Korea | +Gm (10 Mg ha^-1^)  505 kg ha^-1^ | 1112 kg ha^-1^  (120%) | - | - | - | - | 5.2 Mg ha^-1^ | 5.6 Mg ha^-1^  (7.7%) | Lee et al., 2012 |
| Silicate fertilizer (granular) | 2.3  Mg ha^-1^ | 1 year (2008) | Rice (Continuous irrigation) | Typic Haplaquepts  (Clay loam) | South Korea | +Gm (20 Mg ha^-1^)  752 kg ha^-1^ | 1690 kg ha^-1^  (124%) | - | - | - | - | 5.3 Mg ha^-1^ | 5.9 Mg ha^-1^  (11.2%) | Lee et al., 2012 |
| Silicate fertilizer (granular) | 2.3  Mg ha^-1^ | 1 year (2008) | Rice (Continuous irrigation) | Typic Haplaquepts  (Clay loam) | South Korea | +Gm (40 Mg ha^-1^)  1680 kg ha^-1^ | 2300 kg ha^-1^  (37%) | - | - | - | - | 5.16 Mg ha^-1^ | 5.6 Mg ha^-1^  (8.5%) | Lee et al., 2012 |
| Silicate fertilizer (granular) | 2.0  Mg ha^-1^ | 1 year | Rice (Intermittent irrigation) | Typic Haplaquepts  (Clay loam) | South Korea | 16.3 g m^-2^ | 10.8 g m^-2^  (-33.7%) | 45.3 mg m^-2^ | 42.7 mg m^-2^  (-5.7%) | - | - | 4.23 Mg ha^-1^ | 5.15 Mg ha^-1^  (21.7%) | Ali et al., 2015 |
| Revolving furnace slag (RFS) | 2.0  Mg ha^-1^ | 1 year | Rice (Intermittent irrigation) | Typic Haplaquepts  (Clay loam) | South Korea | 16.3 g m^-2^ | 11.9 g m^-2^  (-27%) | 45.3 mg m^-2^ | 41.8 mg m^-2^  (-7.7%) | - | - | 4.23 Mg ha^-1^ | 5.10 Mg ha^-1^  (20.6%) | Ali et al., 2015 |
| Blast furnace slag (BFS) | 2.0  Mg ha^-1^ | 1 year | Rice (Intermittent irrigation) | Typic Haplaquepts  (Clay loam) | South Korea | 16.3 g m^-2^ | 14.7 g m^-2^  (-9.8%) | 45.3 mg m^-2^ | 41.3 mg m^-2^  (-8.8%) | - | - | 4.23 Mg ha^-1^ | 4.78 Mg ha^-1^  (13%) | Ali et al., 2015 |
| Silicate fertilizer (granular) | 2.0  Mg ha^-1^ | 1 year | Rice (Intermittent irrigation) | Sandy loam | Japan | 14.6 g m^-2^ | 12.5 g m^-2^  (-14.4%) | 28.9 mg m^-2^ | 23.8 mg m^-2^  (-17.6%) | - | - | 4.3 Mg ha^-1^ | 5.4 Mg ha^-1^  (25.6%) | Ali et al., 2015 |
| Silicate fertilizer (granular) | 2.0  Mg ha^-1^ | 1 year | Rice (Intermittent irrigation) | Sandy loam | Japan | 14.6 g m^-2^ | +*Azolla* (10 kg pot^-1^) 11.4 g m^-2^  (-21.9%) | 28.9 mg m^-2^ | +*Azolla* (10 kg pot^-1^) 24.5 mg m^-2^  (-15.2%) | - | - | 4.3 Mg ha^-1^ | +*Azolla* (10 kg pot^-1^) 5.9 Mg ha^-1^  (37.2%) | Ali et al., 2015 |
| Silicate fertilizer (granular) | 2.0  Mg ha^-1^ | 1 year | Rice (Intermittent irrigation) | Clay loam | Bangladesh | 15.8 g m^-2^ | 12.9 g m^-2^  (-18.4%) | 39.5 mg m^-2^ | 33.9 mg m^-2^  (-14.2%) | - | - | 4.1 Mg ha^-1^ | 5.25 Mg ha^-1^  (28%) | Ali et al., 2015 |
| Silicate fertilizer (granular) | 2.0  Mg ha^-1^ | 1 year | Rice (Intermittent irrigation) | Clay loam | Bangladesh | 15.8 g m^-2^ | +*Azolla* (10 kg pot^-1^) 11.6 g m^-2^  (-26.6%) | 39.5 mg m^-2^ | +*Azolla* (10 kg pot^-1^) 34.7 mg m^-2^  (-12.2%) | - | - | 4.1 Mg ha^-1^ | +*Azolla* (10 kg pot^-1^) 5.7 Mg ha^-1^  (39%) | Ali et al., 2015 |
| Revolving furnace slag (RFS) | 2.0  Mg ha^-1^ | 3 years  (2000, 2001, 2002) | Rice (Intermittent irrigation) | Sandy loam | Japan | 1255 kg C ha^-1^ | 1084 kg C ha^-1^  (-13.6%) | - | - | - | - | - | - | Furukawa and Inubushi, 2004 |
| Silicate fertilizer (granular) | 2.0  Mg ha^-1^ | 2 years  (2011-2012) | Rice (Intermittent irrigation) |  | China | 2.34 mg m^-2^ h^-1^ | 1.49 mg m^-2^ h^-1^  (-36.3%) | 32.43 µg m^-2^ h^-1^ | 18.62 µg m^-2^ h^-1^  (-42.6%) | - | - | 12.58 Mg ha^-1^ | 12.60 Mg ha^-1^  (0.16%) | Wang et al., 2015 |
| Silicate fertilizer (granular) | 4.0  Mg ha^-1^ | 2 years  (2011-2012) | Rice (Intermittent irrigation) |  | China | 2.34 mg m^-2^ h^-1^ | 1.12 mg m^-2^ h^-1^  (-52.2%) | 32.43 µg m^-2^ h^-1^ | 19.04 µg m^-2^ h^-1^  (-41.3%) | - | - | 12.58 Mg ha^-1^ | 12.84 Mg ha^-1^  (2%) | Wang et al., 2015 |
| Silicate fertilizer (granular) | 8.0  Mg ha^-1^ | 2 years  (2011-2012) | Rice (Intermittent irrigation) |  | China | 2.34 mg m^-2^ h^-1^ | 1.03 mg m^-2^ h^-1^  (-56%) | 32.43 µg m^-2^ h^-1^ | 0.41 µg m^-2^ h^-1^  (-99%) | - | - | 12.58 Mg ha^-1^ | 12.85 Mg ha^-1^  (2.1%) | Wang et al., 2015 |
| Steel slag | 8.0  Mg ha^-1^ | 1 year (2015) | Early rice  (Intermittent irrigation) |  | China | 6.51 g m^-2^ | 5.83 g m^-2^  (-10.4%) | 0.127 g m^-2^ | 0.092 g m^-2^  (-27.5%) | - | - | 4.63 Mg ha^-1^ | 4.67 Mg ha^-1^  (0.8%) | Wang et al., 2018a |
| Steel slag | 8.0  Mg ha^-1^ | 1 year (2015) | Early rice  (Intermittent irrigation) |  | China | Biochar (8.0  Mg ha^-1^) 6.99 g m^-2^ | 4.29 g m^-2^  (-38.6%) | 0.107 g m^-2^ | 0.101 g m^-2^  (-5.6%) | - | - | 4.91 Mg ha^-1^ | 5.06 Mg ha^-1^  (3%) | Wang et al., 2018a |
| Steel slag | 8.0  Mg ha^-1^ | 1 year (2015) | Late rice  (Intermittent irrigation) |  | China | 17.8 g m^-2^ | 15.15 g m^-2^  (-14.9%) | 0.146 g m^-2^ | 0.106 g m^-2^  (-27.4%) | - | - | 6.73 Mg ha^-1^ | 6.83 Mg ha^-1^  (1.5%) | Wang et al., 2018a |
| Steel slag | 8.0  Mg ha^-1^ | 1 year (2015) | Late rice  (Intermittent irrigation) |  | China | Biochar (8.0  Mg ha^-1^) 10.04 g m^-2^ | 11.85 g m^-2^  (18%) | 0.124 g m^-2^ | 0.116 g m^-2^  (-6.5%) | - | - | 6.97 Mg ha^-1^ | 7.2 Mg ha^-1^  (3.3%) | Wang et al., 2018a |
| Steel slag | 8.0  Mg ha^-1^ | 1 year (2015) | Early rice  (Intermittent irrigation) |  | China | - | - | - | - | 13.6 g kg^-1^ | 13.3 g kg^-1^  (-2.2%) | - | - | Wang et al., 2018b |
| Steel slag | 8.0  Mg ha^-1^ | 1 year (2015) | Early rice  (Intermittent irrigation) |  | China | - | - | - | - | + Biochar (8.0  Mg ha^-1^)  18.6 g kg^-1^ | 17.5 g kg^-1^  (-5.9%) | - | - | Wang et al., 2018b |
| Steel slag | 8.0  Mg ha^-1^ | 1 year (2015) | Late rice  (Intermittent irrigation) |  | China | - | - | - | - | 13.4 g kg^-1^ | 16.2 g kg^-1^  (21%) | - | - | Wang et al., 2018b |
| Steel slag | 8.0  Mg ha^-1^ | 1 year (2015) | late rice  (Intermittent irrigation) |  | China | - | - | - | - | + Biochar (8.0  Mg ha^-1^)  16.2 g kg^-1^ | 19.1 g kg^-1^  (18%) |  |  | Wang et al., 2018b |
| Steel slag (granular) | 1.0  Mg ha^-1^ | 1 year  (Dry season) | Rice (Continuous irrigation) | Inceptisol | Indonesia | 135 kg C ha^-1^ | 149 kg C ha^-1^  (10.4%) | 38.75 g N ha^-1^ | 30.57 g N ha^-1^  (-21.1%) | - | - | - | - | Susilawati et al., 2015 |
| Steel slag (granular) | 1.0  Mg ha^-1^ | 1 year  (Wet season) | Rice (Continuous irrigation) | Inceptisol | Indonesia | 335 kg C ha^-1^ | 304 kg C ha^-1^  (-9.3%) | 19.31 g N ha^-1^ | 13.84 g N ha^-1^  (-28.3%) | - | - | - | - | Susilawati et al., 2015 |
| Steel slag (granular) | 2.0  Mg ha^-1^ | 1 year  (Wet season) | Rice (Continuous irrigation) | Inceptisol | Indonesia | 335 kg C ha^-1^ | 299 kg C ha^-1^  (-10.7%) | 19.31 g N ha^-1^ | 12.78 g N ha^-1^  (-33.8%) | - | - | - | - | Susilawati et al., 2015 |
| Steel slag (granular) | 1.0  Mg ha^-1^ | 1 year  (Dry season) | Rice (Continuous irrigation) | Vertisol | Indonesia | 4.99 kg C ha^-1^ | 4.96 kg C ha^-1^  (-0.6%) | 45.99 g N ha^-1^ | 41.43 g N ha^-1^  (-9.9%) | - | - | - | - | Susilawati et al., 2015 |
| Steel slag (granular) | 1.0  Mg ha^-1^ | 1 year  (Wet season) | Rice (Continuous irrigation) | Vertisol | Indonesia | 3.10 kg C ha^-1^ | 2.71 kg C ha^-1^  (-12.6%) | 46.03 g N ha^-1^ | 33.7 g N ha^-1^  (-26.8%) | - | - | - | - | Susilawati et al., 2015 |
| Steel slag (granular) | 2.0  Mg ha^-1^ | 1 year  (Wet season) | Rice (Continuous irrigation) | Vertisol | Indonesia | 3.10 kg C ha^-1^ | 2.52 kg C ha^-1^  (-18.7%) | 46.03 g N ha^-1^ | 28.37 g N ha^-1^  (-38.4%) | - | - | - | - | Susilawati et al., 2015 |
| Calcium silicate | 0.5  Mg ha^-1^ | 2010 | Rice (Continuous irrigation) | Clay loam | Bangladesh | 124 kg ha^-1^ | 93 kg ha^-1^ (-25%) | 0.55 kg ha^-1^ | 0.35 kg ha^-1^  (-36.4%) | - | - | 4290 kg ha^-1^ | 5150 kg ha^-1^ (20%) | Ali et al., 2013 |
| Calcium silicate | 0.5  Mg ha^-1^ | 2010 | Rice (Intermittent irrigation) | Clay loam | Bangladesh | 90 kg ha^-1^ | 61 kg ha^-1^ (-32.2%) | 0.98 kg ha^-1^ | 0.77 kg ha^-1^  (-21.4%) | - | - | 4350 kg ha^-1^ | 5420 kg ha^-1^ (24%) | Ali et al., 2013 |

Values in parenthesis are percentage decrease and/or increase compared to the control. Gm, green manure

**References**

Ali, M.A., Hoque, M.A., and Kim, P. J. (2013). Mitigating global warming potentials of methane and nitrous oxide gases from rice paddies under different irrigation regimes. *Ambio*, *42*(3), 357-368. doi: 10.1007/s13280-012-0349-3

Ali, M.A., Kim, P.J., and Inubushi, K. (2015). Mitigating yield-scaled greenhouse gas emissions through combined application of soil amendments: A comparative study between temperate and subtropical rice paddy soils. *Science of the Total Environment*, *529*, 140-148. [doi: 10.1016/j.scitotenv.2015.04.090](https://doi.org/10.1016/j.scitotenv.2015.04.090)

Ali, M.A., Lee, C.H., Lee, Y.B., and Kim, P.J. (2009). Silicate fertilization in no-tillage rice farming for mitigation of methane emission and increasing rice productivity. *Agric. Ecosyst. Environ*. 132, 16–22. [doi: 10.1016/j.agee.2009.02.014](https://doi.org/10.1016/j.agee.2009.02.014)

Ali, M.A., Oh, J.H., and Kim, P.J. (2008). Evaluation of silicate iron slag amendment on reducing methane emission from flood water rice farming. *Agric. Ecosyst. Environ*. 128, 21–26. [doi: 10.1016/j.agee.2008.04.014](https://doi.org/10.1016/j.agee.2008.04.014)

Furukawa, Y., and Inubushi, K. (2004). Evaluation of slag application to decrease methane emission from paddy soil and fate of iron. *Soil Sci. Plant Nut*. 50, 1029‒1036. doi: 10.1080/00380768.2004.10408570

Gwon, H.S., Khan, M.I., Alam, M.A., Das, S., and Kim, P.J. (2018). Environmental risk assessment of steel-making slags and the potential use of LD slag in mitigating methane emissions and the grain arsenic level in rice (*Oryza sativa* L.). *J. Haz. Mat*. 353:236–243. doi: [10.1016/j.jhazmat.2018.04.023](https://doi.org/10.1016/j.jhazmat.2018.04.023)

Lee, C.H., Kim, S.Y., Villamil, M.B., Pramanik, P., Hong, C.O., and Kim, P.J. (2012). Different response of silicate fertilizer having electron acceptors on methane emission in rice paddy soil under green manuring. *Biol. Fertil. Soils* 48, 435–442. doi: 10.1007/s00374-011-0637-2

Susilawati, H.L., Setyanto, P., Makarim, A.K., Ariani, M., Ito, K., and Inubushi, K. (2015). Effects of steel slag applications on CH_4_, N_2_O and the yields of Indonesian rice fields: a case study during two consecutive rice-growing seasons at two sites. *Soil Sci Plant Nut*. 61, 704-718. [doi: 10.1080/00380768.2015.1041861](https://doi.org/10.1080/00380768.2015.1041861)

Wang, C., Wang, W., Sardans, J., Singla, A., Zeng, C., Lai, D., and Penuelas, J. (2018a). Effects of steel slag and biochar amendments on CO_2_, CH_4_, and N_2_O flux, and rice productivity in a subtropical Chinese paddy field. *Environmental geochemistry and health*, 1-13. doi: 10.1007/s10653-018-0224-7

Wang, W., Lai, D., Abid, A., Neogi, S., Xu, X., and Wang, C. (2018b). Effects of steel slag and biochar incorporation on active soil organic carbon pools in a subtropical paddy field. *Agronomy*, *8*(8), 135. doi:10.3390/agronomy8080135

Wang, W., Sardan, J., Lai, D., Wang, C., Zeng, C., Tong, C., et al. (2015). Effects of steel slag application on greenhouse gas emissions and crop yield over multiple growing seasons in a subtropical paddy field in China. *Field Crops Res*. 171, 146–156. [doi: 10.1016/j.fcr.2014.10.014](https://doi.org/10.1016/j.fcr.2014.10.014)
